# Evaluation of deformity and hand function in cerebral palsy patients

**DOI:** 10.1186/1749-799X-3-52

**Published:** 2008-12-23

**Authors:** Karlen Law, Ellen Y Lee, Boris Kwok-Keung Fung, Lam Shuk Yan, Paata Gudushauri, Kwan Wing Wang, Josephine Wing-Yuk Ip, Shew Ping Chow

**Affiliations:** 1 Occupational Therapy Department, Duchess of Kent Children's Hospital, 12 Sandy Bay, Pok Fu Lam, Hong Kong; 2 Division of Hand and Foot Surgery, Department of Orthopaedics and Traumatology, Queen Mary Hospital, 102 Pokfulam Road, Hong Kong

## Abstract

**Background:**

A cross-sectional study was performed to describe the upper limb deformity and function in cerebral palsy patients and to determine the correlation of deformity, spasticity, motor control, and sensation to hand function in the said population.

**Materials and methods:**

Thirty patients satisfying our inclusion criteria underwent physical, sensory, and functional assessment using a standard protocol. Physical assessment included documentation of the degree of spasticity, deformity and muscle control. Sensation was tested using static two-point discrimination test and stereognosis test. Melbourne Assessment of the Unilateral Upper Limb Function Test (MAULF), Functional Hand Grip Test (FHGT), and Functional Independence Measure for children (WeeFIM) were used to evaluate hand function. Deformity, spasticity, motor control, and sensation were analyzed for correlation with hand function using Pearson Correlation analysis. A p-value of less than 0.05 was considered statistically significant.

**Results:**

Functional deficits of the hand increased with increasing severity of deformity and spasticity. Tetraplegics were most affected by spasticity, deformity, poor motor control, sensory and functional deficits. Triplegics, followed by diplegics had more functional upper limbs in terms of the MAULF and FHGT scores. Unilaterally affected patients (triplegics and hemiplegics) scored better in performance of activities of daily living. The MAULF and FHGT had a stronger correlation to deformity, spasticity and motor control compared to the WeeFIM.

**Conclusion:**

The degree of deformity, spasticity, sensory deficit, and motor control affected the hand function of a cerebral palsy patient significantly. The MAULF and FHGT more accurately represents hand function deficit in cerebral palsy patients.

## Background

Cerebral palsy patients with upper limb involvement have difficulty in performing coordinated movements against spasticity [1]. Performances of hand tasks in these patients require gross and fine hand motion coordinated with visual perception and postural control to enable them to reach, grasp, release and manipulate objects.

Recent published works on cerebral palsy were focused on the management of upper extremity deformity and spasticity [2-6]. Though various assessment tools had been used to assess hand function in this population [3,7-10], review of literature showed no specific evaluation protocol assessing the relationship between the deformity and its projected function. Functional assessment designed by House et al. [3] was simple, but did not fully characterize hand function of cerebral palsy patients. The Melbourne Assessment of the Unilateral Upper Limb Function (MAULF) and Quality of Upper Extremity Skills Test (QUEST) had high inter-rater reliability and construct validity [11,12].

The main objective of this paper was to describe the upper limb deformity and function in cerebral palsy patients with upper extremity involvement, based on the topographic area involved – diplegia, hemiplegia, triplegia, and tetraplegia, using an evaluation protocol developed by our Occupational Therapy Department. Our minor objectives were as follows: (1) to present an objective and representative assessment tool for evaluating hand function in the said population and (2) to evaluate how deformity, spasticity, sensation, and motor control in cerebral palsy patients affect hand function in activities of daily living.

This comprehensive documentation of upper limb deformity and hand function in cerebral palsy patients would allow us to better understand a difficult problem in a more global perspective and help us plan subsequent treatments to improve hand function.

## Methods

### Study population

From 2002 to 2004, 116 cerebral palsy patients aged 5 years or older with preserved visual and auditory senses were screened at the Neuromuscular Clinic of the Duchess of Kent Children's Hospital at Sandy Bay in Hong Kong. Patients with monoplegia, developmental hand age of greater than 16 years assessed using the Bruininks-Oseretsky (B.O.) Test [13] and the "Chopsticks Manipulation Test" (CMT) [14], severe mental retardation based on their Intelligence Quotient (IQ), and those who did not give informed consent were excluded from the study. Forty-three patients were recruited and 30 patients (70%) were present to complete the assessment protocol.

There were 16 females and 14 males aged 6 to 33 years old (mean age: 12.48 years). There were 8 diplegics (26.7%) with an average age of 7.53 years old, 9 hemiplegics (30%) with a mean age of 10.04 years, 3 triplegics (10%) with an average age of 12.53 years, and 10 tetraplegics (33.3%) with a mean age of 21.47 years. Seventy percent had the spastic type of cerebral palsy. (Table 1) 66.7% had normal intelligence. (Table 2) Fifty percent had bilateral involvement while 27% and 23% had left and right-sided involvement, respectively. The study population's developmental hand age ranged from 5.57 years old to 10.13 years old. Results of the 2 tests for developmental (B.O. test) and functional (CMT) hand age were similar in all groups of patients with different topographic involvement.

**Table 1:** Distribution table of the study population in terms of type of cerebral palsy and topographic area of involvement

n = 30	Monoplegia	Diplegia	Hemiplegia	Triplegia	Tetraplegia	Total

Spastic	0	7	7	1	6	21 (70.0%)

Athetoid	0	1	0	0	1	2 (6.70%)

Ataxic	0	0	1	0	0	1 (3.30%)

Dystonic	0	0	0	2	3	5 (16.7%)

Unknown	0	0	1	0	0	1 (3.30%)

Total	0	8	9	3	10	

**Table 2:** Intelligence level of the study population

n = 30	Diplegia	Hemiplegia	Triplegia	Tetraplegia	Total
Normal IQ	5	6	2	7	20(66.7%)

Mild MR	2	2	1	1	6 (20.0%)

Moderate MR	1	1	0	2	4 (13.3%)

Severe MR	0	0	0	0	0 (0.00%)

Profound MR	0	0	0	0	0 (0.00%)

### Study Procedure

Our Occupational Therapy Department developed a structured assessment protocol for the Upper Limb Cerebral Palsy Clinic. This protocol had 4 parts: (1) Physical Assessment, (2) Sensory Assessment, (3) Developmental Hand Assessment, and (4) Hand Function Assessment (Table 3). The developmental hand assessment was performed as described above, on all potential subjects, to exclude those with developmental hand age of greater than 16 years.

**Table 3:** Assessment protocol for cerebral palsy patients developed by occupational therapy department

Four part assessment	Components		
Physical assessment	Contractures	Deformity	Muscle tone

Sensory assessment	Static 2 point discrimination	Stereognosis	

Developmental hand assessment	B.O. test	Chop Sticks Manipulation Test	

Functional hand assessment	Melbourne Assessment of the unilateral upper Limb Function (MAULF) Test	Functional Hand Grip Test (FHGT)	Functional Independence Measure for children (WeeFIM)

Physical Assessment included classification of deformities, assessment of muscle tone and motor control. Classification of typical contracture and deformity was done in anatomic parts using the following: Gschwind & Tonkin [2] for the forearm, Zancolli et al. [15] for the hand and wrist, and House et al. [3] for thumb deformities. Muscle tone was assessed using the Modified Ashworth Scale of Spasticity [16]. Motor control was evaluated using the Zancolli Spastic Hand Evaluation [15], House Functional Classification [3] and Green Functional Classification [9].

Sensory Assessment involved evaluation of static two-point discrimination (2-pd) and stereognosis. Static 2-pd was tested using the Baseline Aesthesiometer. Stereognosis was assessed using the Stereognosis kit (Beechfield Healthcare, Dublin Ireland).

Hand Function Assessment involved (1) evaluation of functional discrepancy from normal subjects using the Melbourne Assessment of the Unilateral Upper Limb Function Test (MAULF) [7] and Functional Hand Grip Test (FHGT) [9] and (2) evaluation of the subjects' performance in activities of daily living (ADLs) using the Functional Independence Measure for Children (WeeFIM, UB Foundation Activities Inc. 2000).

Two occupational therapists evaluated the study population using this protocol in a cross-sectional study. Evaluation was focused on the more severely affected upper limb of each patient.

### Presentation of Results and Data Analysis

The study population was grouped based on topographic area of involvement (diplegic, hemiplegic, triplegic, tetraplegic) for presentation of descriptive results. Descriptive parameters were presented in terms of rank and percentages. Our assessment protocol focused on deformity, sensation, spasticity, motor control and hand function. The correlation between hand function and the other 4 parameters (deformity, sensation, spasticity and motor control) was determined by computing for the correlation coefficient (r) using Pearson Correlation analysis. A p-value of less than 0.05 was considered statistically significant.

## Results

### Physical Assessment

#### Spasticity based on Modified Ashworth Scale

Diplegics, hemiplegics and triplegics had relatively similar levels of spasticity. The 3 groups had increased tone in the tested muscle groups with the majority scoring 1 or 1+. Tetraplegics, with scores of 2 or 3, had marked increase in muscle tone over a wider range of motion. Pronator teres (PT) was the most commonly affected upper limb muscle, regardless of the limb involvement. The Adductor Pollicis (ADP) and Flexor Pollicis Brevis (FPB) were the least involved. The intrinsic muscles were less spastic than extrinsic muscles across all patient groups. (Table 4)

**Table 4:** Results of Modified Ashworth Scale of the affected upper limb

Mean grading n = 30	Diplegia (n = 8)	Hemiplegia (n = 9)	Triplegia (n = 3)	Tetraplegia (n = 10)
Biceps	1+	1	1+	1+

Brachioradialis	1+	1+	1+	3

Pronator Teres	1+	1+	2	3

Flexor Carpi Ulnaris	1+	1+	1+	2

Flexor Digitorum Superficialis	1	1	1	2

Flexor Digitorum Profundus	1	1	1+	2

Adductor Pollicis	1	1+	1+	1+

Flexor Pollicis Brevis	0	1	1+	1+

Lumbricals	1	1+	1+	1+

#### Deformity and Motor Control

Tetraplegics had the most severe deformities and the worst motor control. Even though diplegics, hemiplegics and triplegics have similar levels of spasticity, the hemiplegics had a slightly more severe deformity and worse motor control when compared to the other two groups. (Table 5 and Table 6)

**Table 5:** Upper limb deformity in different topographic groups

Average Grading (↑ Severity 1 → 4) n = 30	Diplegia (n = 8)	Hemiplegia (n = 9)	Triplegia (n = 3)	Tetraplegia (n = 10)
Tonkin's Scale of forearm deformity	1	2	1	2

Zancolli's Scale of hand & wrist deformity	1	1	1	2

House's scale of thumb deformity	1	2	1	3

**Table 6:** Motor control of the upper limbs in different topographic groups

Average Grading n = 30	Diplegia (n = 8)	Hemiplegia (n = 9)	Triplegia (n = 3)	Tetraplegia (n = 10)
Zancolli's Spastic Hand Evaluation (↑ Severity 1 → 4)	2	3	2	3

House's Functional Classification (↓ Severity 0 → 8)	7	5	7	4

Green's Functional Classification (↑ Severity 1 → 4)	2	3	2	3

### Sensory Assessment

Tetraplegics had deficits in both stereognosis (9.7) and 2-pd (6.86 mm). The hemiplegic group had slightly subnormal results in the stereognosis test (8.11). There were no sensory deficits in both diplegic and triplegic groups. (Table 7)

**Table 7:** Results of stereognosis and 2-point discrimination tests of the affected upper limb

n = 30	Diplegia (n = 8)	Hemiplegia (n = 9)	Triplegia (n = 3)	Tetraplegia (n = 7)
Stereognosis (0–13)	11.25	8.11	12	9.7

2PD	4.17 mm	4.57 mm	3.67 mm	6.86 mm

### Hand Function Assessment

The triplegic group (MAULF 89.87%, FHGT 85.13%) performed best in the upper limb function assessment, followed by the diplegic (MAULF 87.96%, FHGT 74.26%), hemiplegic (MAULF 72.77%, FHGT 61.53%) and lastly, the tetraplegic group (MAULF 48.84%, FHGT 46.93%). (Table 8)

**Table 8:** Results of developmental and functional hand assessment (Ax)

	Mean chronological age	Developmental Hand Ax		Functional Ax		ADL Ax
		
n = 30		B.O. test	CMT	MAULF	FHG	WeeFIM
Diplegia	7.53 yr.	5.57 yr.	5.57 yr.	87.96%	74.26%	55.55%

Hemiplegia	10.04 yr.	6.00 yr.	5.72 yr.	72.77%	61.53%	77.96%

Triplegia	12.53 yr.	8.60 yr.	8.60 yr.	89.87%	85.13%	97.09%

Tetraplegia	21.47 yr.	10.13 yr.	9.80 yr.	48.84%	46.93%	42.75%

**Figure 1:**
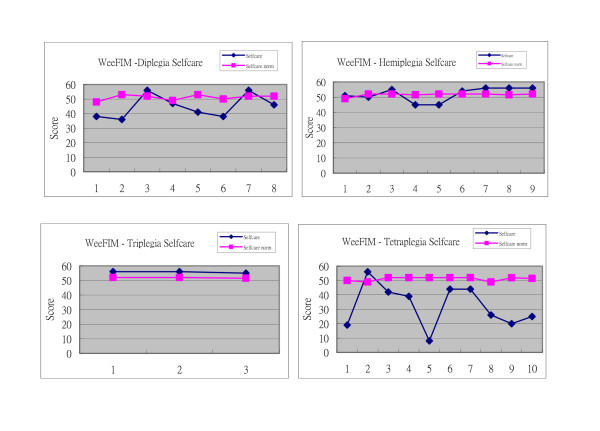
**The self-care score of the WeeFIM in different topographic groups**.

The triplegics, with unilateral hand involvement, scored the highest in the WeeFIM assessment. Hemiplegics scored better than the diplegics by 22.41% in the WeeFIM (Table 8). Cerebral palsy patients with unilateral involvement (hemiplegia and triplegia) could perform as well or better than people without cerebral palsy in ADLs, as measured by their WeeFIM Quotient when compared to normal subjects. (Figure 1)

### Correlation of parameters with MAULF and FHGT

MAULF and FHGT scores had strong correlation with the all parameters (Table 9). Among all deformities, thumb contracture showed the strongest correlation with the functional performance (MAULF: r = -0.775, p = 0.000; FHGT: r = -0.662, p = 0.00). A severe thumb deformity was associated with poorer hand function for both the MAULF and FHGT.

**Table 9:** Correlation of MAULF and FHGT with deformity, spasticity, motor control and sensation

n = 30	MAULF		FHGT	
	
	r	p	r	p
Deformity				

Tonkin's Scale of forearm deformity	-0.671*	0.000	-0.611*	0.000

Zancolli's Scale of hand & wrist deformity	-0.626*	0.000	-0.543*	0.002

House's scale of thumb deformity	-0.775*	0.000	-0.662*	0.000

Spasticity				

Biceps	-0.452*	0.012	-0.442*	0.014

Brachioradialis	-0.530*	0.003	-0.521*	0.003

Pronator Teres	-0.563*	0.001	-0.560*	0.001

FCU	-0.629*	0.000	-0.543*	0.002

FDS	-0.741*	0.000	-0.626*	0.000

FDP	-0.680*	0.000	-0.599*	0.000

ADP	-0.701*	0.000	-0.640*	0.000

FPB	-0.684*	0.000	-0.622*	0.000

Lumbricals	-0.539*	0.002	-0.571*	0.001

Motor Control				

Zancolli's spastic hand evaluation				

House's functional classification	-0.568*	0.001	-0.643*	0.000

Green's functional classification				

Sensation				

Stereognosis	0.422*	0.020		

2 point discrimination	-0.440*	0.036		

For individual muscle spasticity, the flexor digitorum superficialis (FDS) showed the highest correlation with the MAULF (r = -0.741, p = 0.000) while the adductor pollicis (ADP) demonstrated the highest correlation with the FHGT (r = -0.662, p = 0.000).

Sensory deficits showed a statistically significant correlation with the MAULF, but not with the FHGT (Table 10). In the MAULF, 2-pd results had a correlation coefficient (r) of 0.422 (p = 0.002), while that for stereognosis was 0.440 (p = 0.036).

**Table 10:** The correlation between sensory deficit and functional hand assessment

n = 30	Melbourne Assessment of Unilateral Upper limb Function		Functional Hand Grip Test	
	
	Stereognosis	2pd	Stereognosis	2pd
Coefficient of Variation	0.422*	0.440*	0.315	0.393

Significance	0.020	0.036	0.091	0.063

### Correlation of 4 parameters with WeeFIM Quotient

Increasing severity of deformity, sensory deficit, spasticity and motor control were related to decreased hand function in ADLs, as indicated by the decreasing WeeFIM Quotient (Table 11). Hand and wrist deformity (r = -0.541, p = 0.002), spasticity of the PT (r = -0.503, p = 0.005) and FDS (r = -0.601, p = 0.000), and sensory deficit as measured by 2-pd (r = -0.519 p = 0.011) were associated with a lower score.

**Table 11:** Correlation of WeeFIM quotient with deformity, spasticity, motor control and sensation

n = 30	WeeFIM Quotient	
	
	r	p
Deformity		

Tonkin's Scale of forearm deformity		

Zancolli's Scale of hand & wrist deformity	-0.541*	0.002

House's scale of thumb deformity	-0.432*	0.017

Spasticity		

Biceps	-0.384*	0.036

Brachioradialis	-0.409*	0.036

Pronator Teres	-0.503*	0.005

FCU	-0.401*	0.025

FDS	-0.601*	0.000

FDP	-0.495*	0.005

ADP		

FPB		

Lumbricals		

Motor Control		

Zancolli's spastic hand evaluation		

House's functional classification	-0.362*	0.049

Green's functional classification		

Sensation		

Stereognosis		

2 point discrimination	-0.519*	0.011

## Discussion

The upper limb deformity and function in patients with cerebral palsy was described based on the topographic area of involvement. There was an association between the degree of spasticity and motor control with the diplegic group having the mildest spasticity and best motor control, while tetraplegics were the most spastic with poorest motor control. Although the triplegics had slightly more severe spasticity than the hemiplegics, the triplegics scored better in the House's and Green's Functional Classifications for motor control. A possible reason for this could be that the triplegics were older than the hemiplegics in terms of chronological age and developmental hand age (Table 8) in this study, thus they would be better adapted to their condition.

Sensory deficits reflected in the 2-pd & stereognosis tests were dominant in hemiplegics in a previous report [17] but this was not evident in our results. Sensory deficit was only noted in tetraplegics in the current study.

Age is an important determinant of hand function. Normally, hand function develops until the age of 14 then plateaus. Accommodation and fine motor skills improve with age, and then deteriorate during old age. The tetraplegics in our study had the highest developmental hand age (Table 8) and based on this alone, should have been the most functional of the 4 groups. However, this group performed the poorest in all functional assessments (Table 8). This implied that hand function was not dependent on development of fine motor skills alone. Any deformity, spasticity, sensory deficit and impairment in motor control would significantly affect hand function.

The clinical features of cerebral palsy – spasticity, deformity, sensory deficit and poor motor control – were most dominant in the tetraplegics. This group also had the most pronounced chronologic -developmental hand age gap (Table 12). Thus, hand function was most affected in this group of patients.

**Table 12:** Chronological – Developmental Hand Age Gap

	Mean chronological age	Developmental Hand Ax		Max. discrepancy
			
n = 30		B.O. test	CMT	
Diplegia	7.53 yr.	5.57 yr.	5.57 yr.	-1.96 yr. (-35.19%)

Hemiplegia	10.04 yr.	6.00 yr.	5.72 yr.	-4.32 yr. (-75.52%)

Triplegia	12.53 yr.	8.60 yr.	8.60 yr.	-3.93 yr. (-45.70%)

Tetraplegia	21.47 yr.	10.13 yr.	9.80 yr.	-11.67 yr. (-119.08%)

The WeeFIM was a comprehensive tool for assessing upper extremity functional deficits in ADLs. Due to their milder degree of spasticity, diplegics were expected to have the best performance in the ADLs. Similarly, the hemiplegics performed better than the tetraplegics. However, the triplegics were found to be the most functional of the 4 groups with WeeFIM quotients comparable to normal subjects. (Figure 1) Better function in the triplegic, as well as hemiplegic groups, could be brought about by the one-handed technique using the unaffected upper limb they adopted during the assessment. The diplegic group score was affected by involvement of both upper limbs to a much lesser extent in some patients.

To determine the effect of treatment, a simple standardized test to assess hand function deficit was needed. Unlike the comprehensive WeeFIM, the MAULF was an objective test that was easy to accomplish. The specific instructions of the MAULF were better suited to cerebral palsy patients who had problems with motor coordination and postural control.

The WeeFIM was a global function assessment that tested for the ability to perform ADLs. The patient performed certain ADLs and was allowed to use both hands. The unaffected hand could compensate for the affected side, giving the false impression that the affected hand's function was better. The MAULF tested one hand at a time, such that a unilaterally affected patient would not be able to compensate. An improvement in the MAULF score would indicate better function due to treatment and not because of adaptation of the unaffected limb.

The MAULF (r = -0.626, p = 0.000) and FHGT (r = -0.543, p = 0.002) had stronger correlation to hand and wrist deformity than the WeeFIM quotient (r = -0.541 p = 0.002). However, hand sensation did not correlate significantly with FHGT as it did with MAULF. One should bear in mind though that the components of the MAULF could not be analyzed individually, hence it would reflect the patient's level of dysfunction but not the etiology of dysfunction.

Review of current literature showed no standardized assessment battery for assessing hand function of cerebral palsy patients. This study is limited by a low recruitment rate of 70%. Thirty percent of guardians who initially gave consent were not present during the appointed evaluation date. They eventually withdrew their consent on follow-up, stating that the evaluation process would take too much time away from the patient's schooling and other daily activities. The development of a more concise and simpler evaluation score was considered as the direction for future studies by the authors.

This protocol was utilized to meet the study's objectives of generating a comprehensive description of upper limb deformity and hand function in the present population. For practical evaluation of hand function in cerebral palsy patients, the authors recommend the use of the Melbourne Assessment of the Unilateral Upper Limb Function (MAULF) and the Functional Hand Grip Test (FHGT) for reasons previously discussed. Further studies using MAULF and FHGT for evaluating patients before and after surgical treatment are needed to determine their use in monitoring treatment outcome for this population.

## Conclusion

The degree of deformity, spasticity, sensory deficit, and motor control affected the hand function of a cerebral palsy patient significantly. There was a pronounced chronological – developmental and functional hand age gap in all groups. The MAULF and FHGT were strongly correlated to deformity, sensory deficit, spasticity and motor control; making them more representative assessment tools for evaluation of hand function in the said population.

## Consent

Informed consent for publication was obtained from patients or their guardians (in case of minors) during enrollment into the study.

## Abbreviations

MAULF: The following are abbreviations used in the text: Melbourne Assessment of the Unilateral Upper Limb Function Test; FHGT: Functional Hand Grip Test; QUEST: Quality of Upper Extremity Skills Test; 2-pd: two-point discrimination; ADLs: Activities of daily living; WeeFIM: Functional Independence Measure for Children; IQ: Intelligence Quotient; B.O. Test: Bruininks-Oseretsky Test; CMT: Chopsticks Manipulation Test; PT: Pronator teres; ADP: Adductor Pollicis; FPB: Flexor Pollicis Brevis; FDS: Flexor digitorum superficialis; FCU: Flexor carpi ulnaris; FDP: Flexor digitorum profundus; r: Correlation coefficient.

## Competing interests

The authors declare that they have no competing interests.

## Authors' contributions

KL carried out concept design, patient recruitment and follow-up, data collection and analysis, and manuscript writing. EYL carried out literature search, data analysis, manuscript writing and critical revision. BKKF carried out concept design, literature search, patient follow-up, review and approval of the manuscript. LSY, PG, KWW carried out data collection, patient follow up, data analysis and manuscript writing. WYI carried out patient recruitment, review and approval of the manuscript. SPC carried out concept design, patient recruitment, review and approval of the manuscript. All authors read and approved the final manuscript for publication.

## References

[B1] Flett PJ (2003). Rehabilitation of spasticity and related problems in childhood cerebral palsy. J Paediatr Child Health.

[B2] Gschwind C, Tonkin MA (1992). Surgery for cerebral palsy: Part 1. Classification and operative procedures for pronation deformity. J Hand Surg.

[B3] House JH, Gwathmey FW, Fidler MO (1981). A dynamic approach to the thumb-in-palm deformity in cerebral palsy. J Bone Joint Surg.

[B4] Tonkin MA, Gschwind C (1992). Surgery for cerebral palsy: Part 2. Flexion deformity of the wrist and fingers. J Hand Surg.

[B5] Tonkin M, Hatrick NC, Eckersley JR, Couzens G (2001). Surgery for cerebral palsy Part 3: Classification and operative procedures for thumb deformity. J Hand Surg.

[B6] Van Heest E, House H (1999). Upper extremity surgical treatment of cerebral palsy. J Hand Surg.

[B7] Randall MJ, Johnson LM, Reddihough DS (1999). The Melbourne Assessment of Unilateral Upper Limb Functional Test Administration Manual.

[B8] DeMatteo C, Law M, Russell D, Pollock N, Rosenbaum P, Walter S (1992). QUEST: Quality of Upper Extremity Skills Test.

[B9] Eliasson AC, Ekholm C, Carlstedt T (1998). Hand function in children with cerebral palsy after upper-limb tendon transfer and muscle release. Dev Med Child Neurol.

[B10] Arner M, Eliasson AC, Nicklasson S, Sommerstein K, Hägglund G (2008). Hand Function in Cerebral Palsy. Report of 367 Children in a Population-Based Longitudinal Health Care Program. J Hand Surg.

[B11] Cusick A, Vasquez M, Knowles L, Wallen M (2005). Effect of rater training on reliability of Melbourne Assessment of Unilateral Upper Limb Function scores. Dev Med Child Neurol.

[B12] DeMatteo C, Law M, Russell D, Pollock N, Rosenbaum P, Walter S (1993). The reliability and validity of Quality of Upper Extremity Skills Test. Phys Occup Ther Pediatr.

[B13] Bruininks H (1978). Bruininks-Oseretsky Test of Motor Proficiency.

[B14] Chen HM, Chang JJ (1999). The skill components of a therapeutic chopstick task and their relationship with hand function tests. Kaohsiung J Med Sci.

[B15] Zancolli EA, Goldner LJ, Swanson AB (1983). Surgery of the spastic hand in cerebral palsy: Report of the Committee on Spastic Hand Evaluation. J Hand Surg.

[B16] Bohannon RW, Smith MB (1987). Inter-rater reliability of a modified Ashworth scale of muscle spasticity. Phys Ther.

[B17] Van Heest E, House J, Putnam M (1993). Sensibility Deficiencies in the hands of children with spastic hemiplegia. J Hand Surg.

